# The incidence of first-line antiretroviral treatment changes and related factors among HIV-infected sex workers in Nairobi, Kenya

**DOI:** 10.11604/pamj.2017.28.7.10885

**Published:** 2017-09-05

**Authors:** Frank Ndaks Ndakala, Julius Otieno Oyugi, Margaret Ng’wono Oluka, Joshua Kimani, Georg Martin Norbert Behrens

**Affiliations:** 1 University of Nairobi, Institute of Tropical and Infectious Diseases (UNITID), Nairobi, Kenya; 2 State Department of Science and Technology, Directorate of Research Management and Development (DRMD), Nairobi, Kenya; 3 University of Manitoba, College of Medicine, Department of Medical Microbiology, Winnipeg, Manitoba-Canada; 4 University of Nairobi, College of Health Sciences, School of Pharmacology and Pharmacognosy, Nairobi, Kenya; 5 Hannover Medical School, Department of Clinical Immunology and Rheumatology, Hannover, Germany; 6 German Centre for Infection Research, Germany

**Keywords:** Adverse drug reactions, incidence, cART, sex workers

## Abstract

**Introduction:**

In many settings, several factors including adverse drug reactions and clinical failure can limit treatment choices for combined antiretroviral therapy (cART). The aim of the study was to describe the incidence of first-line cART changes and associated factors in a cohort of Kenyan sex workers.

**Methods:**

This was a retrospective review of medical records collected from 2009 to 2013. The review included records of HIV-infected patients aged ≥ 18 years, who received either stavudine or zidovudine or tenofovir disoproxil fumarate-based regimens. Using systematic random sampling, the study selected 1 500 records and censoring targeted the first incident of a drug change from the first-line cART.

**Results:**

The overall incidence rate of cART changes was 11.1 per 100 person-years within a total follow-up period of 3 427.9 person-years. Out of 380 patients who changed cART, 370 (97%) had a drug substitution and 10 (3%) switched regimens. The most commonly cited reasons for changing cART were adverse drug reactions (76%). Tenofovir disoproxil fumarate had a lower drug change rate (1.9 per 100 person years) compared to stavudine (27 per 100 person years). Using zidovudine as the reference group, stavudine-based regimens were significantly associated with an increased hazard of drug changes (adjusted hazards ratio 10.2; 95% CI: 6.02-17.2).

**Conclusion:**

These findings suggest a moderate incidence of cART changes among sex workers in Nairobi, Kenya. Individuals using stavudine were at a higher risk of experiencing a change in their cART, mostly presenting within 20 months, and primarily due to adverse drug reactions.

## Introduction

The pressing need to scale-up combined antiretroviral therapy (cART) in resource-poor settings has resulted in a growing number of patients receiving simple standardised doses of the first-line regimens [1]. A majority of the 24.0 to 28.7 million people infected with human immunodeficiency virus (HIV) live in the resource-poor setting of sub-Saharan Africa [2]. Kenya has the fourth-largest HIV epidemic in the world with over 1.6 million HIV-infected people. Out of 880 000 adults eligible for cART, 656 359 Kenyans are on cART [3]. In spite of the current deficit in treatment with cART, several factors remain a hindrance to the improvement of long-term availability of HIV treatment in Kenya.

Factors such as adverse drug reactions (ADRs), clinical failure, non-adherence to treatment, and concomitant treatment, contribute to the change of the original first-line cART [4-6]. According to the World Health Organization (WHO), the burden of ADRs and regimen changes in sub-Saharan Africa is further complicated by limited antiretroviral treatment choices [7]. In these settings, patients stay on the first-line regimens as long as possible [8, 9]. This is because consecutive regimens are expensive, less effective and patients may not last long on them due to new possible side effects [9]. Studies conducted in developed countries recorded median time that patients stay on a first-line regimen as 1.6 years and 1.8 years [10, 11]. In the Eastern African region, a higher median time to ART change of 3.1 years was recorded in Kenya [12]. In addition, separate studies conducted in Kenya and Ethiopia revealed incidence rates of 18.6/100 person-years (PY) and 10.11/100 PY respectively [13, 14].

Other factors that were observed to influence the change of first-line regimens included WHO stage, low CD4 count, patient’s weight and treatment failure [9, 13]. Since clinicians do not conduct routine viral load measurements to guide the diagnosis of treatment failure, HIV-infected patients in Kenya frequently encounter delayed cART drug changes and are at an increased risk of viral resistance. As a result, the overall financial cost of cART to HIV-infected people could increase, thereby jeopardising long-term prognosis. Due to these limitations, designing strategies aimed at prolonging the use of the original cART regimen among the less privileged HIV-infected populations is essential. However, there is insufficient information that can inform such strategies. This study describes the incidence of and factors influencing the first-line cART regimen changes in a cohort of adult Kenyan sex workers.

## Methods

### Ethical considerations

The study received ethical approval from Kenyatta National Hospital-University of Nairobi-Ethics Review Committee. The sex workers outreach programme (SWOP) administration granted access to the electronic and hard copy medical files in the clinics.

### Setting

The study setting was at the sex workers outreach programme (SWOP) clinics in Nairobi, Kenya. The SWOP cohort of sex workers consists of female commercial sex workers and men who have sex with men (MSM) for commercial reasons. The SWOP primary health care facilities offer treatment to HIV-infected sex workers who live in Nairobi slums. The University of Manitoba from Canada and the University of Nairobi jointly manage the SWOP clinics. Since the start of the SWOP facilities, about 15 000 HIV-infected people have been initiated to cART. The SWOP sites selected for the study were in proximity to each other to enable sampling of patients from the same population.

### Study design and population

This was a retrospective analysis of electronic medical records of HIV-1 infected sex workers receiving cART in five SWOP clinics. The study characterised the cohort as cART-naive adult sex workers aged ≥ 18 years, who started the first-line cART between January 2009 and December 2013 and had a minimum of one follow-up visit record. The SWOP clinics started cART for people with severe or advanced HIV clinical disease (WHO clinical stage III or IV) and in people with CD4 count ≤ 350 cells/mm^3^. Before 2008, the first-line cART regimens consisted of either stavudine (d4T) or zidovudine (AZT) combined with lamivudine (3TC) and efavirenz (EFV) or nevirapine (NVP). Since 2009, the first-line regimen consisted of AZT or d4T or TDF, with 3TC and either NVP or EFV. However, in 2010, patients already on AZT or d4T containing regimens continued using them unless toxicity developed. All patients had their follow-up visits after two weeks on starting cART, monthly after stabilising and every quarter for monitoring of treatment outcomes. Clinicians measured and reviewed every quarter the CD4 cell counts, haematological, hepatological and renal function defects in patients who had stabilised on cART. Because viral load tests are costly, conducting these tests was rare. Additionally, during each visit, clinicians collected the patients’ details on standardised forms to support data transcription in the Kenyan AIDS Control database.

### Data collection

The study utilised prospectively maintained electronic medical records sourced from Kenya AIDS Control Database. Before extracting electronic files, there was a thorough examination of the information on the medical records. Extraction of any missing information was by referring to the hard copies of medical files accessed from the clinics. Using a simple random sampling technique, the study independently selected about 1 500 medical records from the electronic database as a sampling frame. To conduct a simple systematic random sampling, there was a random selection of the first record from the first four records in the sampling frame and subsequent addition of every fourth record. The study collected independent variables that included demographic and anthropometric characteristics including age, sex, weight (kg) height (m) and body mass index (BMI). Other variables collected in the study were clinical and laboratory variables, including ADRs, CD4 cell counts, pregnancy status, creatinine, alanine aminotransferase (ALT) / aspartate aminotransferase (AST) and haemoglobin (HB); treatment variables, including the type of cART regimen, cART initiation and regimen change dates, reasons for change, new cART regimens, treatment outcomes and dates of outcomes. The clinicians used calibrated scales to measure all the anthropometric measurements. Measurements of current weight (kg) and height (cm) were taken in duplicate based on procedures recommended by WHO [15]. The analysis used the arithmetic means obtained from the two measurements then calculated body mass index (BMI) by dividing the weight in kg by the square of the height in metres.

### Exposure and outcomes

The exposures in the study were age at cART initiation, sex, CD4 cell counts, HB, cART regimen type, and period on cART. The primary outcome during this analysis was the initial change from the first-line antiretroviral treatment characterised as an individual drug substitution involving d4T, AZT, and TDF regimens or a regimen switch to second-line treatment containing a protease inhibitor-based regimen [16]. Censoring occurred at the time of the first incident of a drug change from the first-line cART with the focus on changes connected to d4T, AZT, and TDF. The reasons for a drug change covered adverse drug reactions (ADRs), treatment failure (considered as an immunologic failure or clinical failure or virologic failure, or any combination of the three), and other reasons. The study defined immunological failure as a fall in CD4 cell count to 50% from the on-treatment peak value or persistent CD4 cell count of < 100 cells or fall of CD4 counts to pre-therapy baseline. Patients with clinical failure showed new or recurrent adverse events indicative of WHO stage IV condition after 6 months of effective treatment. Patients with virologic failure showed plasma viral load of > 1 000 copies/ ml based on two consecutive viral load measurements after 3 months, with adherence support. The reasons for drug change classified as other reasons included drug changes occasioned by defaulted treatment, tuberculosis, pregnancy and drug stoke-out. The analysis used the Kenya National Clinical Manual to define ADRs [17]. The manual defines an ADR as any severe and life-threatening effect of antiretroviral (ARV) drugs presented at normal doses. Polyneuropathy consisted of symptoms that included tingling, burning, pain, numbness, weakness in limbs, paraesthesia, muscle weakness, and inability to walk. Features defining lipodystrophy included a hump on the back, loss of facial fat, thinning or wasting of the limbs, and or accumulation of fat at the abdomen. The study defined hepatological disorder based on the amount of liver transaminase enzymes detected in the blood. Hepatological disorder included patients with > 40 IU/L of alanine aminotransferase (ALT) or aspartate aminotransferase (AST). The amount of haemoglobin (HB) present in the blood helped to characterise patients with the haematological disorder. This group of patients consisted of individuals with < 11 g/dl of HB, without a history of anaemia or > 1 g / dl drop in HB after beginning cART [18]. Patients with a rash had erythema, pruritus, and severe mucous membrane involvement (Stevens-Johnson syndrome). Patients with renal abnormality composed of cases with > 150 µmol / l of serum creatinine after beginning cART. Baseline characteristics included measurements of CD4 cells, HB, ALT, AST, weight, height, BMI and creatinine measured closest to cART starting date (within 6 months before up to 1 week after cART initiation).

### Statistical analysis

The study performed data analysis using SPSS statistics version 17.0. Chicago: SPSS Inc. (USA). Medians and interquartile ranges described variables with a skew distribution while percentages described categorical variables. The calculations of drug-specific incident rates were in the form of rates per individuals’ initiating the precise drug. Estimating survival time to first drug change was through Kaplan-Meier survival analysis [19]. Censoring of patients targeted the time of the first incident of a drug change from the first-line cART. Modelling for the risk factors related to drug changes employed the Cox Proportional Hazards analysis. The model covered the following variables: sex, age at cART initiation, height, weight, BMI, HB, CD4 cell count, AST, ALT, creatinine, types of cART regimens and calendar year of cART initiation. The analysis considered significant factors with a p - value of 0.05.

## Results

Out of the eligible 1 500 medical records sampled, the analysis excluded 50 medical records of patients with duplicate data sets. The demographic characteristics of the remaining 1 450 eligible medical records showed that 77.8% of the patients in the study were female commercial sex workers. The rest of the study population consisted of male commercial sex workers (men who have sex with men (MSM)). Overall, most of the patients started combination antiretroviral therapy (cART) in 2009 and 2010 (Table 1). About 38% of the patients started on AZT-based regimens, 36% on d4T-based regimens, and 26% on TDF-based regimens. Compared to patients who started either AZT or TDF-based cART, patients who initiated to d4T containing cART had a lower median CD4 cell count, which reflected a greater likelihood of HIV-1 disease progression.

**Table 1:** Characteristics of HIV-infected patients initiated on ART in SWOP clinics from January 2009-December 2013 (n = 1 450)

Patient characteristics	Subjectson AZT(n=553)	Subjectson TDF(n=375)	Subjectson d4T(n=522)
Age (years)-median [IQR]	38 (32-44)	39 (33-45)	40 (35-46)
**Sex:**			
Female	420 (75.9%)	318 (84.8%)	390 (74.7%)
Median Weight (kg)–median (IQR)	65 (57-73)	65 (58-75)	64 (56-73)
Median Height (m)–median (IQR)	1.6 (1.6-1.7)	1.7 (1.6-1.7)	1.6 (1.6-1.7)
Median BMI–median (IQR)	22.9 (20.7-26.7)	23.5 (20.6-27.9)	23.5 (20.8-27.0)
**NNRTI-Based**			
Efavirenz	127 (23%)	166 (44.3%)	89 (17%)
Nevirapine	426 (77%)	209 (55.7%)	433 (83%)
Baseline CD4 count (µmol/l)–median (IQR)	310 (205.5-412.5)	290 (188-411)	258 (158-367.3)
Baseline HB (g/dl) – median (IQR)	12.7 (11-13.9)	12.4 (10.8-14)	12.8 (11.2-13.9)
Baseline AST-median (IQR)	22 (17-30)	23 (17-31)	24 (19-32)
Baseline ALT-median (IQR)	18.7 (13-27.9)	19 (13-26.9)	21.1 (15-29.8)
Baseline creatinine (µmol/l)-median (IQR)	85 (75-96)	82 (73-97)	87 (75-99)
**Calendar Year**			
2009	219(39.6%)	97(25.9%)	439(84.1%)
2010	100(18.1%)	49(13.1%)	27(5.2%)
2011	146(26.4%)	116(30.9%)	50(9.6%)
2012	88(15.9%)	113(30.1%)	6(1.1%)

IQR-Interquartile range; NNRTI-Non-nucleoside reverse transcriptase inhibitors; AZT-zidovudine; TDF- tenofovir disoproxil fumarate; d4T-stavudine; ALT-alanine transaminase; AST-aspartate aminotransferase; CD4-cluster of differentiation 4; HB-haemoglobin.

### Incidence of first-line cART drug changes

The study observed an overall incident rate of total drug changes of 11.1 per 100 person-years (P/Ys) within a total follow-up period of 3 427.9 person-years (Table 2). Out of 380 patients who changed cART, 370 (97%) had a drug substitution and 10 (3%) switched regimens. Compared to patients using TDF (n = 12) and AZT (n = 25), 63.8% of d4T users (n = 333) substituted regimens, reflecting a higher proportion of total drug changes. Treatment switches from first to second-line drugs accounted for 2.6% (n = 10) of drug changes. The cumulative P/Ys of exposure for patients receiving TDF and AZT-based cART were 783.9 P/Ys and 1 393 P/Ys respectively (Table 2). The rates of drug changes were highest among patients initiating d4T-based regimens [27.0/100 PYs (95% CI: 22.8-27.4)] compared to either AZT [1.9/100 PYs (95% CI: 0.4-2.0)] or TDF [2.0/100 PYs (95% CI: 0.9-4.5)]. Table 3 shows the reasons for the first incident of drug changes from the first-line cART as captured by the clinicians (Table 3). The clinicians cited ADRs as the main reason for a drug change, accounting for 75.5% of the captured drug changes. The overall prevalence rate of ADRs was 34.9%. The prevalence rates of specific ADRs that included lipodystrophy, polyneuropathy, haematological disorder, hepatological disorder, rash and renal abnormality were 41.7% (n = 211), 29.4% (n = 149), 15.4% (n = 78), 9.3% (n = 47), 2.8% (n = 14) and 1.4% (n = 7) respectively. Most patients who substituted d4T had lipodystrophy and polyneuropathy. Twelve patients out of the 78 patients with the haematological disorder had a drug substitution (Table 3). Individual drug substitutions from one first-line to a different first-line regimen were the key drivers of cART drug changes attributable to ADRs. However, the reason cited for treatment switches from a first-line cART regimen to a second-line cART regimen was treatment failure.

**Table 2:** Rates of d4T, AZT and TDF 1st drug changes during follow-up

Rate of Change	Subjects on AZT(n = 553)	Subjects on TDF(n = 375)	Subjects on d4T(n = 522)
Total Time Exposed (PY)	1, 393	783.9	1, 251
Total Drug Changes (%)	26 (4.7%)	16 (4.3%)	338 (64.8%)
Rate of Total Drug Changes (/100 PY)	1.9 (95% CI: 0.4-2)	2.0 (95% CI: 0.9-4.5)	27.0 (95% CI: 22.8-27.4)
Drug Substitutions (%)	25 (4.5%)	12 (3.2%)	333 (63.8%)
Rate of Drug Substitutions (/100 PY)	1.8 (95% CI: 0.9-2.6)	1.5 (95% CI: 0.8-1.9)	26.6 (95% CI: 22.5-27.2)
Regimen Switch (%)	1 (0.2%)	4 (1.1%)	5 (1%)
Rate of Regimen Switch (/100 PY)	0.1 (95% CI: 0.1-0.3)	0.5 (95% CI: 0.2-0.7)	0.4 (95% CI: 0.2-1.6)

IQR-Interquartile range; PY-person years; AZT-zidovudine; TDF- tenofovir disoproxil fumarate; d4T-stavudine

**Table 3:** Reasons for changing treatment from d4T, AZT, and TDF during ART in HIV-infected Kenyan patients

Reasons for drug change	AZT (n=26)	TDF (n=16)	d4T (n=338)
Lipodystrophy	4 (15.4%)	3 (18.8%)	204 (60.4%)
Polyneuropathy	2 (7.7%)	0 (0%)	54 (16%)
Haematological disorder	7 (26.9%)	0 (0%)	3 (0.9%)
Renal abnormality	0 (0%)	0 (0%)	0 (0%)
Hepatological disorder	0 (0%)	1 (6.3%)	6 (1.8%)
Rash	2 (7.7%)	0 (0%)	1 (0.3%)
Treatment failure	1 (3.8%)	3 (18.8%)	9 (2.7%)
Other Reasons	10 (38.5%)	9 (56.4%)	61(18%)

ADRs-adverse drug reactions; ART-antiretroviral therapy; AZT-zidovudine; TDF- tenofovir disoproxil fumarate; d4T-stavudine. Other reasons (pregnancy, tuberculosis, and drug stoke-out).

### Risk factors of antiretroviral drug changes

**Figure 1:**
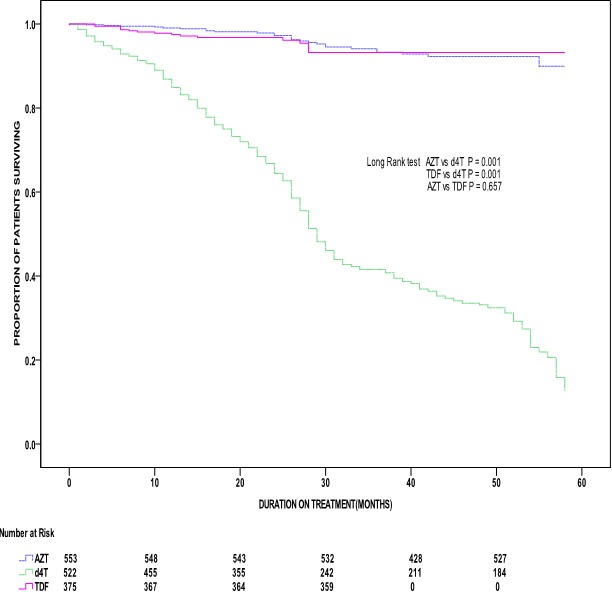
Kaplan-Meier analyses of time to first AZT, d4T and TDF drug change among HIV-infected patients in SWOP clinics

A Kaplan-Meier plot presented in Figure 1 illustrates the time to the first incident of drug changes from the first-line cART stratified by backbone first-line antiretroviral drug regimens (Figure 1). Throughout follow-up time, there was a gradual rise in cART drug changes attributable to ADRs, treatment failure and other reasons that included pregnancy, tuberculosis and defaulted treatment. Since d4T accounted for up to 79.3% of cART drug changes, the survival time on d4T significantly related to a high risk of drug changes compared to TDF and AZT (p < 0.001). However, the graph representing AZT regimen was close to that of TDF regimen, implying the difference in survival time to the first incident of a drug change between the two regimens was insignificant (p = 0.657). After 12 months of cART, the percentage of patients who changed either TDF, AZT or d4T were 0.6%, 0.3%, and 4.6% respectively. However, after 60 months of cART use, about 1.8% of patients on AZT had a drug change compared to 23.3% of d4T users. Further, the study performed both univariate and multivariate analyses of time to the first incident of drug changes from first-line cART (Table 4). In a univariate analysis, an increased hazard of drug changes related to CD4 cell counts of ≥ 200 cells/mm^3^ ((hazards ratio 4.51; 95% CI: 3.63-5.60), creatinine ((hazards ratio 1.01; 95% CI: 1.00-1.01), d4T ((hazards ratio 12.4; 95% CI: 7.51-20.5), and the calendar year 2010 ((hazards ratio 6.24; 95% CI: 2.3-16.9). To introduce BMI, HB, AST and ALT as continuous variables in the Cox model, the SPSS analysis used standardised residuals to check for linearity before transforming the data by Log_10_ transformation. To avoid over-adjustment, the multivariate model did not adjust for height and weight. Instead, after adjusting for BMI, a statistically insignificant increased risk of cART drug changes related to BMI (hazards ratio 1.0; 95% CI: 0.99-1.00), AST (adjusted hazards ratio 1.34; 95% CI: 0.70-2.57) and ALT [adjusted hazards ratio 1.17; 95% CI: 0.66-2.60]. In addition, using AZT as a comparator, d4T regimen independently associated with a significantly increased risk of cART drug changes [adjusted hazards ratio 10.16; 95% CI: 6.02-17.16], while the risk among TDF users was low (adjusted hazards ratio 0.83; 95% CI: 0.44-1.55).

**Table 4:** Cox hazards model of risk factors for duration to first cART drug changes in SWOP clinics

Variable	Univariate analysis			Multivariate analysis		
	Hazard ratio	95% CI	*P*-value	Hazard ratio	95% CI	*P*-value
**Age (years)**						
Age <40	**Reference**					
Age ≥40	0.81	0.66-0.99	**0.04**	0.85	0.69-1.04	0.12
**Sex**						
Female	**Reference**					
Male	0.85	0.67-1.06	0.15	0.95	0.73-1.22	0.66
**Baseline CD4 cell count**						
<200 cells/mm^3^	Reference					
≥ 200 cells/mm^3^	4.51	3.63-5.60	**0.00**	1.00	1.00-1.01	0.68
Baseline weight (Kg)	0.84	0.80-1.00	**0.02**			
Baseline Height (cm)	0.99	0.98-1.00	**0.02**			
Baseline BMI	1.00	1.00-1.00	0.43	1.0	0.99-1.00	0.47
Baseline HB	0.99	0.95-1.02	0.48	0.87	0.29-2.56	0.79
Baseline AST	1.00	0.99-1.00	0.83	1.34	0.70-5.57	0.37
Baseline ALT	1.00	1.00-1.00	0.96	1.17	0.66-2.56	0.60
Baseline creatinine	1.01	1.00-1.01	**0.00**	1.01	1.00-1.01	0.23
**ART Regimen**						
Zidovudine	**Reference**					
Stavudine	12.4	7.51-20.5	**0.00**	10.2	6.02-17.2	**<0.01**
Tenofovir	0.86	0.46-1.59	0.62	0.83	0.44-1.55	0.56
**Calendar Year**						
2009	**Reference**					
2010	6.24	2.30-16.9	**0.00**	1.23	0.44-3.39	0.71
2011	1.61	0.54-4.77	0.39	0.86	0.29-2.57	0.78
2012	1.19	0.89-3.66	0.76	0.57	0.18-1.77	0.33

CD4-Cluster of Differentiation 4; cART-combination antiretroviral therapy; BMI-Body Mass Index; HB-haemoglobin; ALT-alanine transaminase; AST-aspartate aminotransferase; SWOP-sex workers outreach programme

## Discussion

The present findings report an overall incident rate of total drug changes of 11.1 per 100 person-years (P/Ys) within a total follow-up period of 3 427.9 PYs. Specifically, the incident rates of total drug changes for patients on AZT, TDF and d4T were 1.9 per 100 P/Ys, 2.0 per 100 PYs and 27 per 100 PYs respectively. Most patients who substituted d4T had symptoms of lipodystrophy and polyneuropathy. In addition, an increased risk of drug substitution was independently associated with d4T-based regimens (adjusted hazards ratio 10.16; 95% CI: 6.02-17.16).

The overall incident rate of cART drug changes reported in the current study is comparable to findings of other studies conducted in Kenya. Studies conducted in Western Kenya reported incident rates of cART drug changes as 18.6 per 100 PYs and 13.3 per 100 PYs [5, 13]. However, the Swiss cohort study which had a higher incident rate of 41.5 per 100 PYs [20] is indicative of higher incident rates of cART drug changes observed in developed countries [10, 21]. The observed variations in incident rates of cART changes could be due to several reasons including variations in study methodologies, treatment monitoring, and selection bias. In addition, the difference could be because the present study only considered ADRs leading to cART change. Many studies have reported ADRs, as the main reason for changing cART in several resource-poor settings [22, 23]. The current findings support two Kenyan studies that cited cART-related toxicity as the main reason for changing treatment [13, 24]. This study further reports that patients who substituted cART had lipodystrophy and polyneuropathy which accounted for 41.7% and 29.4% of the total ADRs recorded. However, comparisons between these studies remain a challenge because of variations in ADR reporting ways, study periods, types of the cART regimens in use [25, 26] and the use of concurrent medication [27]. There were fewer incident rates connected to the switch of a first-line cART regimen to a second-line regimen, suggesting limited treatment options available in this setting [9]. Alternatively, since there was no data on patients who failed to switch to second-line therapy due to loss to follow-up, this study may have underestimated the number of patients who switched regimens.

Despite the high prevalence of ADRs including lipodystrophy and polyneuropathy, the use of d4T-based cART was common in the SWOP clinics. Compared to AZT and d4T, TDF showed signs of a well-tolerated drug regimen with fewer drug changes. Studies with comparable findings exist in Zambia and South Africa [28-31]. In Uganda and Swaziland, the main causes of d4T-based drug substitutions included lipodystrophy and polyneuropathy, whilst anaemia caused most of the AZT-based drug substitutions [32, 33]. Similarly, the present study had a significantly higher proportion of patients who needed d4T substitutions. Most substitutions were in line with the WHO’s recommendations of 2010 [7]. Though different studies have associated lower rates of TDF substitutions with renal impairment [34, 35], these findings did not confirm the association.

In a multivariate analysis, an increased hazard of drug changes related to the d4T regimen. This further supports evidence in the literature that relates TDF to a good safety record [29, 32, 36]. Older age is a well-known risk factor in patients with polyneuropathy, lipodystrophy and renal toxicity [7]. However, these findings found an insignificant association between older age and an increased hazard of drug substitution. Similar contrasting data exist in the literature, but they have been less consistent [29, 32]. Study limitations included the poor recording of ADRs and inadequate reporting by the health care practitioners. It is possible that some ADRs were mistaken with effects of advanced HIV-1 infection, concomitant medications, or co-morbidities. In addition, by restricting the analysis to the first incident of cART drug changes alone, the study may have underestimated the true number of the overall drug changes. However, this study had a robust data set, with adequate sample size and minimal missing data in the descriptive variables.

## Conclusion

This study reports a moderate incidence of cART changes among HIV-infected sex workers from Nairobi, Kenya. The most common reason identified for changing cART was the presentation of adverse drug reactions. Most individuals using stavudine were at a higher risk of experiencing a change in their cART due to lipodystrophy and polyneuropathy, mostly presenting within 20 months after the start of treatment.

### What is known about this topic

- HIV-infected patients live longer due to increased accessibility to cART;
- There is an increased prevalence of cART-related adverse drug reactions in the general population;
- In the general population, cART-related adverse drug reactions contribute to high rates of cART changes.

### What this study adds

- The prevalence of cART-related adverse drug reaction in a cohort of sex workers in Nairobi, Kenya;
- The incidence rate of cART changes among sex workers in Nairobi, Kenya;
- The most common reason cited for changing cART in this setting.

## Competing interests

The authors declare no competing interest.
